# High consumption of unhealthy commercial foods and beverages tracks across the complementary feeding period in rural/peri‐urban Cambodia

**DOI:** 10.1111/mcn.13485

**Published:** 2023-02-08

**Authors:** Guy‐Marino Hinnouho, Elaine L. Ferguson, Amy MacDougall, Hou Kroeun, Prak Sophonneary, Mary Chea, Alissa M. Pries

**Affiliations:** ^1^ Helen Keller International New York City New York USA; ^2^ London School of Hygiene and Tropical Medicine London UK; ^3^ Ministry of Health Phnom Penh Cambodia

**Keywords:** ultraprocessed foods, unhealthy commercial foods and beverages

## Abstract

Consumption of unhealthy commercial foods and beverages (UCFB) is common among infants and young children living in low‐ and middle‐income countries. Such foods can displace other nutritious foods, however, there is limited evidence on how this consumption tracks across time. This study assessed and tracked UCFB consumption of children living in rural/peri‐urban Cambodia during the complementary feeding period, identified UCFB consumption patterns of these children, and explored the association between UCFB consumption and growth. A 6‐month longitudinal cohort study was implemented among 567 caregivers of children aged 10–14 months at recruitment. UCFB consumption was estimated each month via a telephone‐administered 7‐day food frequency questionnaire, and UCFB consumption patterns were identified based on changes in this frequency of consumption over time. The majority of children either maintained (45.7%, *n* = 246) or developed (43.5%, *n* = 234) an unhealthy consumption pattern and only 10.8% (*n* = 58) of children maintained/transitioned into a healthy consumption pattern. High consumers of UCFB at 10–14 months had a 4.7 (CI: 4.7 [3.1–7.2]) times odds of being high consumers of UCFB at 15–19 months (*p* < 0.001). There was a trend of lower length‐for‐age *z*‐scores (LAZ) among children maintaining or developing an unhealthy consumption pattern (~−0. *SD* LAZ) compared to children maintaining/transitioning into a healthy consumption pattern, however, this association was not statistically significant. Findings indicate that high UCFB consumption begins during infancy and tracks into early childhood. National policies and programmes centred on early interventions addressing the use of UCFB for infant and young child feeding are needed.

## Key messages

- This study found a high frequency of unhealthy commercial food and beverage (UCFB) consumption by children under 2 years of age in rural/peri‐urban Kandal province, Cambodia.
- This high UCFB consumption began during older infancy and tracked into early childhood.
- National attention should focus on designing and implementing early interventions to increase caregivers' awareness of the risks high UCFB consumption poses for older infants and young child nutrition.
- National policies and programmes should address the widespread availability of UCFB and promote increased breastfeeding and consumption of affordable, nutritious, and locally available foods during the complementary feeding period.

## Graphical abstract

This study assessed and tracked unhealthy commercial food and beverage (UCFB) consumption during the complementary feeding period in rural/peri‐urban Cambodia, and explored the association between UCFB consumption and growth. A 6‐month longitudinal cohort study was implemented among 567 caregivers of children aged 10–14 months at recruitment. The majority of children either maintained (45.7%, *n* = 246) or developed (43.5%, *n* = 234) an unhealthy consumption pattern and only 10.8% (*n* = 58) of children maintained/transitioned into a healthy consumption pattern; high UCFB consumers at 10–14 months had nearly five times the odds of being high consumers at 15–19 months.

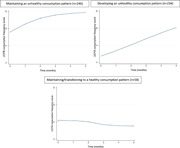

## 1 INTRODUCTION

Ensuring adequate nutrition, in both quantity and quality of the diet, during the first 2 years of a child's life is critical for growth and development (Black et al., 2008). This 24‐month window represents a period of opportunity when the foundations of optimum health, growth and neurodevelopment across the lifespan are established (World Health Organization, 1998). Infants' and young children's nutritional requirements during this period are high to support their rapid growth and development. At 6 months of age, it is recommended to introduce nutrient‐dense and safe complementary foods, together with continued breastfeeding for up to 2 years of age or beyond (World Health Organization & UNICEF, 2003).

The types of foods fed to infants and young children during this complementary feeding period are critical to ensure their nutrient requirements are met. The world has seen increased production and availability of commercially produced ultraprocessed foods and beverages in recent decades (Juul et al., 2022; Monteiro et al., 2013; Wang et al., 2021), resulting in dietary pattern shifts towards substantial increases in the consumption of these products in low‐ and middle‐income countries (LMIC) (Monteiro et al., 2011; Pagliai et al., 2021; Popkin et al., 2012). Commercially produced, highly‐processed foods and beverages are often high in added sugar, salt and unhealthy fats (Luiten et al., 2016; Monteiro et al., 2011), making them inappropriate for infant and young child feeding (IYCF). Consumption of unhealthy commercial foods and beverages (UCFB) is prevalent among both adults and children in many LMICs (Feeley et al., 2016; Green et al., 2019; Huffman et al., 2014; Martins et al., 2013; Pries et al., 2016). For infants and young children, high consumption of such foods during the vital complementary feeding period can displace consumption of nutritious foods, potentially resulting in inadequate intakes of micronutrients (Anderson et al., 2008; Kimmons et al., 2005; Pries, Rehman, et al., 2019) and has been associated with an increased risk of overweight/obesity in later childhood (Jimenez‐Cruz et al., 2010; Rose et al., 2017).

In Cambodia, despite a significant improvement in child health and nutrition status in recent years, including reduced child mortality and child stunting, inadequate and inappropriate IYCF practices leave many children at high risk of malnutrition during the early stages of life (Som et al., 2018). According to the 2022 Cambodia Demographic and Health Survey, stunting, underweight, wasting and overweight affect 22%, 16%, 10% and 4% of children under 5 years of age, respectively (National Institute of Statistics [NIS] [Cambodia] et al., 2022). Moreover, just over half of children 6–23 months of age achieved the recommended minimum dietary diversity of at least five nutritious food groups (National Institute of Statistics [NIS] [Cambodia] et al., 2022), indicating that a high proportion of these children are at risk of not consuming nutritionally adequate diets during the complementary feeding period. Consumption of UCFB among Cambodian infants and young children is prevalent. A 2014 study reported that 38% of children 6–11 months of age and 63% of 12–23 months old in Phnom Penh consumed commercially produced foods or beverages the previous day (Pries et al., 2016), which might contribute to inadequate intakes of nutrients. A 2019 study among urban 12–23‐month‐old Nepalese children found that higher consumption of unhealthy snack foods and beverages (USFB) was associated with lower dietary micronutrient adequacy and length‐for‐age *z*‐scores (LAZ) (Pries, Rehman, et al., 2019).

Although prevalent consumption of UCFB among infants and young children has been noted in several LMICs, there is limited evidence regarding the association between the consumption of these products and infant and young children's nutritional status in LMIC settings (Jannat et al., 2020; Pries, Filteau, et al., 2019; Pries, Rehman, et al., 2019) and whether such consumption tracks over time. Tracking refers to the predictability of a measurement of a given risk factor early in life for values of the same risk factor later in life (Twisk et al., 1997). The period of complementary feeding is important for setting taste preferences and infant attitudes towards foods, and there is evidence to suggest nutritional habits formed in infancy track into childhood and beyond (Birch, 1999; De Cosmi et al., 2017). Thus, frequent consumption of UCFB early in life may increase the likelihood of high consumption of such foods later in life.

The pattern of UCFB consumption among Cambodian young children across the complementary feeding period has not been assessed, and it is not known whether this UCFB consumption is associated with nutritional status. This study, therefore, aimed to (1) assess and track UCFB consumption of children living in rural/peri‐urban Kandal province, Cambodia, over 6 months during the complementary feeding period, (2) identify UCFB consumption patterns during the complementary feeding period, and to (3) explore the association between these UCFB consumption patterns and young children's linear and ponderal growth.

## 2 METHODS

### 2.1 Study design and sampling procedure

Ethical approval for this study was provided by the Cambodian National Ethics Committee for Health Research (no. 104) and the London School of Hygiene and Tropical Medicine (no. 17941 ‐ 3). A household census among the 93 villages in the rural/peri‐urban district of Khsach Kandal, Kandal Province, Cambodia, was conducted before data collection, to enumerate all children within the 10–14 months age range for enrolment. This province was selected because it provides a range of rural and peri‐urban communities, and therefore helps fill a gap in evidence on UCFB consumption in nonurban LMIC settings (Pries, Filteau, et al., 2019). Because prior research in Cambodia has shown that UCFB consumption becomes more prevalent across the complementary feeding period (Pries et al., 2016), this age range was selected for enrolment as it would allow us to track potential increases in both the prevalence and frequency of UCFB consumption from older infancy into early childhood. Due to an outbreak of COVID‐19, the census was stopped after 66 of the 93 villages were completed, covering approximately two‐thirds of the district's population. All the primary caregivers of eligible children who were identified during the census were contacted by telephone to be part of the study. Primary caregivers were defined as the person responsible for caring for the child during the day, including the person who is mainly responsible for feeding the child. If more than one eligible child per household was identified during the census, then one child was randomly sampled for enrolment using Stata 15 (StataCorp). Children were excluded if they were severely ill on the day of the interview, if they had a congenital/physical malformation that inhibited feeding, or if the child was a resident of the district less than 3 of the 6 months before enrolment.

A 6‐month longitudinal cohort study was implemented from June 2021 through January 2022. Verbal informed consent was obtained from all primary caregivers before enrolment. The study was conducted across six timepoints, with data collected monthly. Data collection was conducted via a telephone interview, to ensure participant safety during the COVID‐19 pandemic. At each timepoint, a structured questionnaire was first administered to the primary caregiver, followed by a 7‐day food frequency questionnaire (FFQ) to assess their child's food/beverage consumption. After each interview, caregivers were provided phone credit to compensate them for their time. All tools for this survey were translated into Khmer, back‐translated to English to ensure accuracy, and pretested before data collection to ensure participant comprehension. Because of the outbreak of COVID‐19, anthropometric measurements were not taken at baseline. By the end of the 6‐month follow‐up period, the COVID‐19 situation had improved, and anthropometric measurements of the child and the child's mother were taken at the family's home.

### 2.2 Sample size

To detect a 0.5 standard deviation (SD) difference in LAZ and weight‐for‐length *z*‐score (WLZ) between UCFB consumption pattern groups, a sample size of 309 (*n* = 103 per pattern group) was necessary (*α* = .05, 1−*β* = .8). Anticipating a high loss to follow‐up of approximately 30%, a total of 540 children were enroled in the survey. A final sample size of approximately 378 children at timepoint 6 was determined adequate to assess associations between UCFB consumption patterns across the 6 months of follow‐up and growth outcomes.

### 2.3 Tools and data collection procedures

An interviewer‐administered structured questionnaire was used to collect information on demographic and socioeconomic characteristics pertaining to the caregiver (age, educational attainment, marital status, religion, employment status), to the household (asset ownership, housing materials, access to utilities and food security) and to the child (sex, age, breastfeeding status, morbidity, birthweight, immunisation and deworming status). Information on breastfeeding practices, morbidity and food security was collected at each timepoint, and birthweight, immunisation and deworming status were collected at timepoint 6. A measure of social desirability was also collected at timepoint 6, using a 13‐question module adapted from Reynolds' short forms of the Marlow‐Crowne social desirability scale (Reynolds, 1982). Social desirability bias is the tendency of survey respondents to answer questions in a manner that will be viewed favourably by others (Krumpal, 2013). The consumption of UCFB may be considered unfavourable, therefore we assessed whether reported consumption of UCFB varied by levels of social desirability score.

A 7‐day interviewer‐administered FFQ was used to ask caregivers the number of days in the previous week the child consumed unhealthy commercial packaged/branded foods and beverages. The FFQ consisted of nine categories of commercially packaged foods and beverages: (1) sweet biscuits/crackers, (2) savoury crisps/crackers, (3) bakery items (cake, doughnuts, sponge cake), (4) confectionary items (candy, sweets, chocolate), (5) soft drinks, (6) sweet milk, (7) juice drinks, (8) malt/chocolate drinks and (9) instant noodles. These categories of foods were based on sentinel unhealthy foods, and beverages identified as inappropriate for young children by the recently updated 2021 World Health Organization (WHO) IYCF indicators (World Health Organization & UNICEF, 2021), and have been identified as commonly consumed by Cambodian infants and young children in prior research (Pries et al., 2017). Data were collected electronically on tablets using the open‐source online platform ONA (Ona Systems) and the Open Data Kit application. Completed questionnaires were submitted to the ONA platform daily, and the database was downloaded and stored securely.

Before anthropometric measurements, caregiver‐child pairs were first screened for COVID‐19 using the WHO interim guidance for public health surveillance of COVID‐19 (World Health Organization, 2020) and only participants who were not suspected, not probable, not confirmed or not contact cases of COVID‐19 were measured. Anthropometric measurements were taken by two trained anthropometry staff using standardised procedures (Cogill, 2003). Maternal and child weight were measured on a frequently calibrated SECA digital scale (±100 g precision; model 874; SECA). Child recumbent length was measured to ±0.1 cm precision using a length board (Portable baby/child L‐hgt mea.syst/SET‐UNICEF) and maternal height to ±0.1 cm precision using a stadiometer (SECA 213). Duplicate measurements were taken, and the mean of the two measurements was used in the analysis. If the two measurements differed by more than 1 cm for adult height, more than 0.5 cm for child length or more than 0.5 kg for weight, the measurements were discarded, and two more were taken. The anthropometry team was standardised before data collection following WHO procedures (De Onis et al., 2004). The mean technical error of measurement for length was 0.13 and was considered acceptable.

### 2.4 Data analysis

To track UCFB consumption across the complementary feeding period, each child's consumption of UCFB was calculated at each timepoint  based on the frequency of consumption of UCFB in the week before interview. From the weekly FFQ, a UCFB consumption score was generated for each child based on how many of the 7 days in the prior week each of the nine categories of unhealthy commercial packaged/branded foods/beverages were consumed was calculated, with a possible range of 0–63. From these scores, terciles were created to identify low, moderate and high‐frequency consumers of UCFB at each timepoint. Two methods were then used to track UCFB consumption across the 6 months within the complementary feeding period assessed. First, proportions of children with a change in tercile category or those with a stable tercile category between timepoints 1 and 6 were estimated, and Cohen's Kappa (Κ) (Cohen, 1960) between low/moderate versus high tercile of frequency of consumption of UCFB at these timepoints was calculated. A *Κ* < 0.20 represents a poor agreement, *Κ* of 0.21–0.40 a fair agreement, *Κ* of 0.41–0.60 a moderate agreement, *Κ* of 0.61–0.80 a good agreement and *Κ* > 0.81 a very good agreement (Landis & Koch, 1977). Second, a logistic regression model was used to explore the odds of being a high consumer of UCFB at timepoint 6 (15–19 months) when a child was a high UCFB consumer at timepoint 1 (10–14 months).

To identify UCFB consumption patterns across the complementary feeding period, the repeated measures of UCFB consumption scores were modelled and summarised using a series of Poisson regressions to estimate the slopes and intercepts for each participating child. A separate Poisson regression was fitted for each individual, which estimated the slope and intercepts on the log scale, for that individual. The slopes were exponentiated and then divided into ‘decreasing’ (decrease by more than 10% over time), ‘stable’ (less than 10% change over time) and ‘increasing’ (more than 10% increase over time). The median of the intercepts was 5.5, and intercepts were divided into two equal‐sized groups defined as low (frequency of consumption of UCFB ≤ 5.5) and high (frequency of consumption of UCFB > 5.5). Based on the intercept and the change in slope over time, children were then grouped into one of three UCFB consumption patterns: (1) maintaining/transitioning into a healthy consumption pattern across 6 months (holding a consistently low UCFB score/moving from high to low UCFB score over time), (2) developing an unhealthy consumption pattern across 6 months (moving from a low to high UCFB score over time) and (3) maintaining an unhealthy consumption pattern across 6 months (holding a consistently high UCFB score over time). The characteristics of the participants were presented as means ± *SD* or medians with interquartile range or proportions, as appropriate, by UCFB consumption patterns. We used analysis of variance to compare sociodemographic characteristics between UCFB consumption patterns and Bonferroni post hoc tests to test for specific differences between consumption patterns for continuous variables and the *χ* ^2^ test for dichotomous measures. Reported *p* values are two‐tailed and *p* < 0.05 were considered to be statistically significant.

The association between UCFB consumption patterns across the 6 months of follow‐up and growth at timepoint 6 was explored using linear regression models. The primary growth outcomes of interest were LAZ and WLZ, which were calculated based on WHO 2006 growth standards (WHO Multicentre Growth Reference Study Group, 2006). Models were further adjusted for covariates associated (*p* < 0.05) with LAZ (low birthweight, breastfeeding status, caregiver age, caregiver relation to child, caregiver's education level and household wealth index) and WLZ (caregiver age, caregiver relation to the child, breastfeeding status and household wealth index) in bivariate models. Principal components analysis was applied to data related to asset ownership, utility access and house crowding to derive a wealth index (Vyas & Kumaranayake, 2006). Households were then grouped in wealth terciles as a proxy for household socioeconomic status. Food security was defined using the Household Food Insecurity Access Scale (HFIAS), and households were categorised as food secure or food insecure (mild/moderate/severe) (Coates et al., 2007). A social desirability score was generated based on the sum of socially desirable answers out of the 13‐question module; a score of 13 was the highest social desirability score. All analyses were carried out using Stata 15. Sensitivity analyses were conducted for missing data, where key characteristics were compared between subjects with data and subjects with missing data at each time point and differences were assessed between the two groups.

## 3 RESULTS

**Figure 1:**
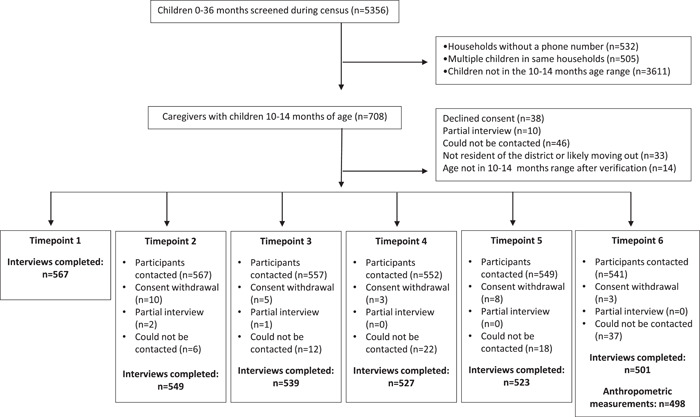
Flowchart of participant recruitment, exclusion and missing data at each timepoint.

Results from the sampling procedure are detailed in Figure 1. Of the 708 caregivers with children 10–14 months of age who were contacted for enrolment, 141 (19.9%) were excluded for the following reasons: 46 could not be contacted, 14 children were not in the 10–14 months age range after verification, 33 were not resident of the district or were likely moving out before the end of the study, 38 declined consent and 10 partial interviews were conducted. A final sample of 567 caregivers was interviewed at timepoint 1. Over the course of the 6‐month follow‐up period, 29 (5.1%) participants dropped out of the study, and between 3.2% and 5.6%, depending on the timepoint, could not be reached. A total of 498 children had anthropometric data at endline. The mean age of children at enrolment was 11.9 ± 1.2 months and 17.4 ± 1.1 months at endline.

Sociodemographic characteristics of participating children, caregivers and households by UCFB consumption patterns are presented in Table 1. Children who maintained an unhealthy consumption pattern were, on average, 0.6 months older than children in the other two UCFB consumption patterns (*p* < 0.001). Just over half of the children (56.0%) were still breastfeeding at enrolment, with breastfed children receiving an average of 10 breastfeeds on the day before the interview. At timepoint 6, when children were 15–19 months of age, 11.7% (*n* = 58) were stunted and 5.0% (*n* = 25) were wasted. Caregivers were 34.5 years old on average; the majority of them were mothers of the children (74.2%), 15.4% were grandmothers, 6.3% were fathers, 2.4% were aunts and 1.6% were grandfathers. Nearly half of the caregivers (48.0%) had attended at least a secondary school level of education. A significantly higher proportion of caregivers of children maintaining/transitioning into a healthy consumption pattern had attended at least a secondary level of education compared with caregivers of children maintaining an unhealthy consumption pattern (63.8% vs. 43.1%; *p* = 0.016). No other characteristics were statistically different between the UCFB consumption pattern groups.

**Table 1:** Sociodemographic characteristics by UCFB consumption patterns.^a^

		Unhealthy commercial foods/beverages consumption patterns			
	All children (*n* = 538)^b^	Maintaining unhealthy consumption pattern (*n* = 246)	Developing unhealthy consumption pattern (*n* = 234)	Maintaining/transitioning into a healthy consumption pattern (*n* = 58)	*p*
*Child characteristics*					
Age in months, mean ± *SD*	11.9 ± 1.2	12.2 ±1.2^a^	11.6 ± 1.2^b^	11.6 ± 1.1^b^	<0.0001
Female sex, *n* (%)	266 (49.4)	127 (51.6)	111 (47.4)	28 (48.3)	0.645
Ever breastfed, *n* (%)	509 (94.6)	236 (95.9)	217 (92.7)	56 (96.6)	0.236
Breastfed in last 24 h, *n* (%)	144 (56.0)	126 (50.8)	16 (47.1)	17 (60.7)	0.540
Morbidity in last 2 weeks, *n* (%)^c^	200 (37.2)	102 (41.5)	82 (35.2)	16 (27.6)	0.100
Low birthweight (<2.5 kg)^d^	35 (7.0)	15 (6.5)	17 (7.9)	3 (5.9)	0.808
Fully immunised, *n* (%)^e^	456 (96.6)	207 (95.4)	200 (97.6)	49 (98.0)	0.398
Anthropometric status^f^					
LAZ	−0.79 ± 1.1	−0.84 ± 1.3	−0.80 ± 1.0	−0.52 ± 1.1	0.200
Stunting (LAZ <−2)	58 (11.7)	24 (10.4)	29 (13.4)	5 (9.8)	0.572
WLZ	−0.39 ± 1.0	−0.36 ± 1.0	−0.41 ± 1.0	−0.39 ± 1.0	0.906
Wasting (WLZ <−2)	25 (5.0)	11 (4.8)	12 (5.5)	2 (3.9)	0.872
*Caregiver characteristics*					
Mother of child, *n* (%)	399 (74.2)	185 (75.2)	173 (73.9)	41 (70.7)	0.775
Age in years, mean ± *SD*	34.5 ± 11.4	34.0 ± 10.7	34.6 ± 11.9	36.1 ± 12.0	0.239
Attended at least a secondary level of education, *n* (%)	258 (48.0)	106 (43.1)^a^	115 (49.2)^a^ ^,^ b	37 (63.8)^b^	0.016
Currently employed, *n* (%)	230 (42.8)	105 (42.7)	99 (42.3)	26 (44.8)	0.941
Works outside the home, *n* (%)	134 (24.9)	62 (25.2)	59 (25.2)	13 (22.4)	0.909
*Household characteristics*					
Food secure	125 (23.2)	51 (20.7)	59 (25.2)	15 (25.9)	0.449
Lowest wealth index	178 (33.2)	94 (38.4)	67 (28.8)	17 (29.3)	0.198

Abbreviations: ANOVA, analysis of variance; LAZ, length‐for‐age *z*‐score; UCFB, unhealthy commercial foods and beverages; WLZ, weight‐for‐length *z*‐score.

^a^ All characteristics assessed at baseline except for birthweight, immunisation and anthropometric data, which were collected at the final timepoint; labelled values in a row without a common letter differ, *p* < 0.05; Mixed‐effects Poisson regression model used to summarise repeated measures of UCFB into maintaining an unhealthy consumption pattern, developing an unhealthy consumption pattern, maintaining/transitioning into a healthy consumption pattern. ANOVA and Bonferroni post hoc test were used to compare differences between groups.

^b^ *n* = 29 removed from analysis.

^c^ Morbidity in last 2 weeks included experiences of fever, cough or diarrhoea.

^d^ *n* = 497.

^e^ *n* = 472.

^f^ *n* = 498.

**Figure 2:**
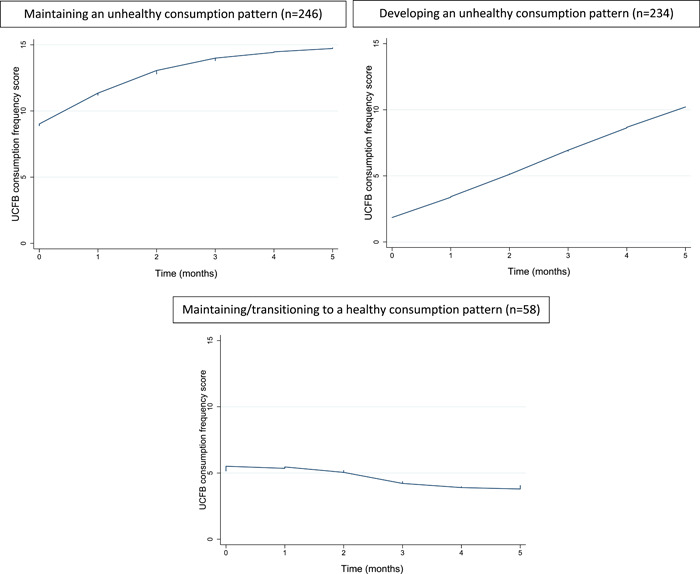
Unhealthy commercial foods and beverages consumption patterns of older infants and young children in Kandal province, Cambodia. UCFB, unhealthy commercial foods and beverages.

During the 6‐month follow‐up period, the majority of children either maintained (45.7%, *n* = 246) or developed (43.5%, *n* = 234) an unhealthy consumption pattern, and only 10.8% (*n* = 58) of children maintained a healthy consumption pattern/transitioned from an unhealthy consumption pattern to a healthy one (Figure 2).

Children's frequency of consumption of UCFB across the 6 timepoints is detailed in Table 2. The proportion of children who consumed any UCFB in the previous week increased steadily from 81.7% (*n* = 463) at 10–14 months of age to 97.4% (*n* = 488) at 15–19 months of age. In addition, the median number of times UCFB were consumed in the previous week increased from 4 at 10–14 months to 11 at 15–19 months and the median number of UCFB categories consumed in the previous week increased from 2 at 10–14 months to 4 at 15–19 months. The most common UCFB category consumed across all age groups was sweet milk, which increased from 3 days per week at 10–14 months to 7 days per week at 15–19 months. We found evidence of social desirability bias for reported consumption of UCFB; a one‐point increase in social desirability score was associated with a 0.41‐point decrease in UCFB consumption score at timepoint 6 (*β* [95% CI] = −.41 [−0.74, −0.07]; *p* = 0.017). Tracking of children in the highest UCFB consumption tercile was fair when comparing timepoint 1 and timepoint 6 (Cohen's *Κ* = 0.33). In addition, high consumers of UCFB at 10–14 months had 4.7 (CI: 4.7 [3.1–7.2]) times the odds of being high consumers of UCFB at 15–19 months (*p* < 0.001).

**Table 2:** Children's frequency of consumption of UCFB across the 6 timepoints.^a^

	Timepoint 1 (*n* = 567)	Timepoint 2 (*n* = 549)	Timepoint 3 (*n* = 539)	Timepoint 4 (*n* = 527)	Timepoint 5 (*n* = 523)	Timepoint 6 (*n* = 501)
Age in months, mean ± *SD*	11.9 ± 1.2	12.8 ± 1.2	13.9 ± 1.2	15.0 ± 1.2	16.0 ± 1.2	17.4 ± 1.1
Consumption of UCFB in last 7 days, *n* (%)	463 (81.7)	474 (86.3)	482 (89.4)	478 (90.7)	492 (94.1)	488 (97.4)
Times UCFB consumed in last 7 days, median [IQR]	4 [1–8]	7 [3–11]	8 [3–13]	10 [5–15]	10 [5–15]	11 [7–16]
UCFB categories consumed in last 7 days, median [IQR]	2 [1–3]	2 [1–4]	3 [1–4]	3 [2–5]	3 [2–5]	4 [3–5]
Days consumed in the last 7 days						
Sweet biscuits/crackers	2 [2–4]	3 [2–5]	3 [2–5]	3 [2–5]	3 [2–5]	3 [2–4]
Savory crisps/crackers	2 [1–3]	3 [2–4]	3 [2–4]	3 [2–5]	3 [2–4]	3 [2–4]
Bakery items	1 [1–2]	2 [1–2]	2 [2–3]	2 [1–3]	1 [1–2]	1 [1–3]
Confectionery items	1 [1–2]	2 [1–3]	2 [1–3]	2 [1–3]	2 [1–3]	2 [1–3]
Soft drinks	1 [1–2]	2 [1–3]	2 [1–3]	2 [1–3]	2 [1–3]	2 [1–3]
Sweet milk	3 [2–7]	5 [2–7]	7 [2–7]	7 [3–7]	7 [3–7]	7 [3–7]
Malt/chocolate drinks	1 [1–2]	2 [1–3]	2 [1–3]	1 [1–2.5]	1 [1–2]	3 [2–3]
Juice drinks	1 [1–2]	2 [1–2]	2 [1–2]	2 [1–3]	2 [1–3]	1 [1–2]
Instant noodles	1 [1–2]	1 [1–2]	1 [1–2]	1 [1–2]	1 [1–2]	1 [1–2]

Abbreviations: FFQ, food frequency questionnaire; IQR, interquartile range; UCFB, unhealthy commercial foods and beverages.

^a^ Values are presented as mean ± *SD*, *n* (%) or median [IQR].

There was a marginal association between children maintaining an unhealthy consumption pattern and LAZ (*p* = 0.076), with children who maintained an unhealthy consumption pattern having a LAZ 0.31 *SD* lower as compared to children who maintained/transitioned into a healthy consumption pattern (Table 3). However, this marginal association was attenuated after adjustment for low birthweight, breastfeeding status, caregiver age, caregiver relation to child, education and household wealth index. In both unadjusted and adjusted models for breastfeeding status, caregiver age, caregiver relation to the child, and household wealth index, there was no evidence that UCFB consumption patterns were associated with WLZ (all *p* > 0.05 with coefficients close to zero).

**Table 3:** Association between UCFB consumption patterns and growth.

		Unadjusted		Adjusted^a^, ^b^	
	n^c^ (%)	*β* (95% CI)	*p*	*β* (95% CI)	*p*
*LAZ*					
Maintaining/transitioning into healthy consumption pattern	51 (10.2)	Ref.		Ref.	
Developing an unhealthy consumption pattern	217 (43.6)	−0.28 (−0.63; 0.07)	0.112	−0.21 (−0.54; 0.12)	0.212
Maintaining an unhealthy consumption pattern	230 (46.2)	−0.31 (−0.66; 0.03)	0.076	−0.23 (−0.57; 0.10)	0.171
*WLZ*					
Maintaining/transitioning into healthy consumption pattern	51 (10.2)	Ref.		Ref.	
Developing an unhealthy consumption pattern	217 (43.6)	−0.01 (−0.31; 0.29)	0.939	0.00 (−0.28; 0.29)	0.981
Maintaining an unhealthy consumption pattern	230 (46.2)	0.03 (−0.27; 0.33)	0.849	0.06 (−0.23; 0.34)	0.697

*Note*: Mixed effects Poisson regression model used to summarise repeated measures of UCFB and linear regression models used to assess association with LAZ and WLZ; UCFB.

Abbreviations: LAZ, length‐for‐age *z*‐score; UCFB, unhealthy commercial foods and beverages; WLZ, weight‐for‐length *z*‐score.

^a^ LAZ adjusted for low birthweight, breastfeeding status, caregiver age, caregiver relation to child, caregiver's education level and household wealth index.

^b^ WLZ adjusted for caregiver age, caregiver relation to child, breastfeeding status and household wealth index.

^c^ Total *n* = 498.

## 4 DISCUSSION

### 4.1 High consumption of UCFB and types of UCFB consumed

In this longitudinal cohort study, we found that UCFB were frequently consumed by children under 2 years of age in rural/peri‐urban Cambodia. Similar results of high UCFB consumption have been shown in other LMIC settings, including Brazil and Nepal, but primarily in urban locations (Karnopp et al., 2017; Nogueira et al., 2022; Pries, Rehman, et al., 2019). Our results also showed that a significant number of infants were not breastfed at enrolment. This finding is alarming given the importance of breastfeeding in reducing the risk of childhood infections and premature mortality as well as minimising nutrition‐related harm to cognitive development (Horta et al., 2015; Victora et al., 2016).

In our study, sweet milk, sweet biscuits/crackers and savoury crisps/crackers were the commercial beverages and foods most often consumed, with sweet milk being consumed daily, on average, once children had reached 12 months of age. This finding is worrisome because previous research has reported that frequent intake of sugar‐sweetened beverages (SSB) at 10–12 months of age significantly increased the likelihood of having dental caries at 6 years (Park et al., 2015) and there is evidence to suggest that early childhood carries are associated with lower WLZ (Turton et al., 2022). High consumption of these ultraprocessed, commercially produced foods and beverages can contribute to higher intakes of added sugar, sodium and unhealthy fats, and they are also often nutrient‐poor, which increases the risks of inadequate intakes of nutrients. Therefore, global guidance (World Health Organization & UNICEF, 2021) and several national dietary guidelines (Healthy Ireland., 2018; Ministerio de Salud MINSA Panama, 2018; U.S. Department of Agriculture & U.S. Department of Health and Human Services, 2020) recommend avoiding their use for IYCF.

### 4.2 Tracking consumption of UCFB

To the best of our knowledge, this is the first study in an LMIC to show high consumption of UCFB early in life tracking across the complementary feeding period. This finding is consistent with studies from high‐income countries (Lioret et al., 2013; Luque et al., 2018; Rose et al., 2017). In a study among Australian young children under 2 years of age, the proportion who consumed SSB and sweet, energy‐dense snacks in the preceding day increased by 3.5 and 2.6 times, respectively, at 18 months compared to 9 months (Lioret et al., 2013). Similarly, American children with a dietary pattern characterised by foods high in energy density, such as SSB and sweet desserts at 9 months of age, had a higher frequency of intake of these foods at 6 years of age (Rose et al., 2017) and European children who were the highest consumers of added sugars and unhealthy fats at 2 years of age had a 3.6 greater odds of also being the highest consumers of these foods at 8 years of age (Luque et al., 2018). Together, these results suggest that early introduction of UCFB might contribute to overconsumption of UCFB later in childhood, presumably through the influence of taste development and food preferences early in life on food preferences later in life (Stein et al., 2012; Ventura & Mennella, 2011). High consumption of these products early in life is associated with increased odds of overweight/obesity in childhood (Jimenez‐Cruz et al., 2010; Rose et al., 2017), which can increase the risk of noncommunicable diseases, such as diabetes and cardiovascular diseases (Costa et al., 2018; Malik et al., 2013; Neri et al., 2022). In the context of increasing overweight/obesity (Nakphong & Beltrán‐Sánchez, 2021), our findings raise concerns regarding childhood dietary quality and its implications for overnutrition as Cambodia undergoes a nutrition transition.

### 4.3 Association with linear and ponderal growth

We observed a nonsignificant trend of a lower LAZ among children maintaining or developing an unhealthy consumption pattern (~−0.3 *SD* LAZ or −0.2 *SD* LAZ in an adjusted model) as compared to children maintaining/transitioning into a healthy consumption pattern; whereas there was no relationship between UCFB consumption patterns and WLZ. Our study, however, was underpowered to detect a 0,2‐0.3 *SD* difference in LAZ between pattern groups (our study sample required 103 children per consumption pattern group to detect a 0.5 SD, but only 58 children were observed maintaining/transitioning into a healthy consumption pattern). Our results are consistent with a previous study, in urban Nepal, showing LAZ, but not WLZ was significantly lower among 12–23 month‐old children in the highest compared with lowest terciles of dietary energy from unhealthy foods and beverages (0.29 *SD* lower) (Pries, Rehman, et al., 2019). In this Nepalese study, there was no difference in energy intakes among high and low consumers of UCFB, indicating that excessive energy intake from these foods was not occurring (Pries, Rehman, et al., 2019). While our Cambodian study did not measure dietary energy intakes, the lack of association between UCFB consumption patterns and WLZ suggests that, similar to Nepal, high UCFB consumption, did not result in excessive energy intakes. Together, these findings suggest that higher consumption of these products may be associated with poor linear growth among young children in areas where displacement of nutritious foods by UCFB can result in poor dietary adequacy. Further research on the mechanisms through which these UCFB are contributing to undernutrition is needed, particularly in LMIC settings.

### 4.4 Importance of healthy eating habits early in life

Healthy eating habits are developed early in life (Schwartz et al., 2011), making it important to avoid feeding infants and young children UCFB. In contexts where micronutrient gaps exist in the complementary feeding diets of infants and young children (Beal et al., 2021), overconsumption of these food/beverage products can displace more nutritious foods, resulting in reduced dietary adequacy with consequent impairments in child growth (Anderson et al., 2008; Pries, Rehman, et al., 2019). In Cambodia, where just over 50% of children 6–23 months consume diets achieving recommended minimum dietary diversity (National Institute of Statistics [NIS] [Cambodia] et al., 2022), diet displacement of nutrient‐dense foods by UCFB is a significant concern. In addition, given the establishment of eating habits in these early years, the dietary patterns observed among young children in this study may also indicate an increased risk for overnutrition in later childhood by predisposing them to UCFB consumption throughout childhood. A longitudinal study among infants and young children in the United States found that infants whose dietary patterns were characterised by energy‐dense, unhealthy foods at 9 months continued to have a high consumption of such foods at 6 years of age and were more likely to be overweight (Rose et al., 2017).

### 4.5 Drivers of choice for UCFB

Several factors that influence caregivers' feeding decisions for UCFB have been identified in previous research. Marketing is a known driver for purchasing and consuming UCFB products (Sadeghirad et al., 2016). Despite calls from the WHO to restrict the marketing of foods and nonalcoholic beverages to children (WHO, 2010), the marketing of these products has expanded rapidly in many LMIC contexts, especially in the Asia region (Kelly et al., 2016), including Cambodia (Pries et al., 2017). Further research on the role of marketing on infant and young child UCFB consumption specifically is needed, and regulations that restrict the marketing of these foods to children and caregivers are essential. A child's preference for UCFB products is another factor known to influence how caregivers feed their children (Green et al., 2019; Pries et al., 2017; Rahman et al., 2016; Sharma et al., 2019), including Cambodian mothers of children 6–23 months of age (Pries et al., 2017). Added sugar, salt, fats and other additives in UCFB create hyper‐palatable products (Gibney et al., 2017); in contexts where feeding styles respond to child demands, this palatability can lead to poor nutritional outcomes (Anzman et al., 2010). To reduce the consumption of UCFB and improve IYCF and nutritional status, caregiver awareness of the nutritional quality of these products needs to be increased. Our findings and prior research indicate that intervening early to safeguard child diets is vital. A recent trial in rural Bangladesh reported significantly lower consumption of SSB and unhealthy snack foods, as compared to a control group, among young children whose mothers participated in a nutrition education programme beginning in the second trimester of pregnancy and continuing throughout the complementary feeding period (Jannat et al., 2020). Similar benefits may occur, in this rural/peri‐urban Cambodian context, from educational strategies that highlight the risks of introducing nutrient‐poor foods that are high in added sugar and salt to children early in life. In addition, caregivers could be encouraged to feed locally available nutrient‐rich foods as snacks during the complementary feeding period to improve child health, growth and development and reduce risks of obesity later in life.

### 4.6 Limitations

This study has several limitations. While we were able to successfully implement a telephone survey within the context of COVID‐19, this design prevented the collection of quantitative dietary intake data to quantify UCFB consumption and their contribution to overall energy and nutrient intakes, as well as overall dietary adequacy. In addition, we were not adequately powered to detect a statistically significant difference in growth outcomes between UCFB consumption patterns. Another limitation of this study is the lack of anthropometric measurements at baseline, due to the COVID‐19 pandemic, which prevented us from adjusting for LAZ and WLZ differences.

## 5 CONCLUSION

This study found that UCFB were frequently consumed by children under 2 years of age in rural/peri‐urban Khsach Kandal, Cambodia. This consumption began during late infancy and tracked into early childhood. UCFB can displace nutritious foods in the diet and increase the risk of inadequate dietary intakes of nutrients that are important for child growth, health and development. It may also lead to the development of unhealthy consumption patterns that contribute to overweight/obesity later in life. What is first consumed early on can influence what children prefer and what caregivers feed for months or years after. Therefore, drivers of feeding practices need to be better understood and caregivers equipped with knowledge and skills to enable optimal complementary feeding. To reduce overconsumption of UCFB among Cambodian infants and young children, caregivers' exposure to the marketing of these products should be reduced, and interventions implemented to increase caregivers' awareness of the risks high consumption of UCFB poses for young child nutrition. Furthermore, policies and programmes are needed that encourage the consumption of healthy, nutritious, locally available foods during the complementary feeding period. Finally, additional research in LMIC settings undergoing a nutrition transition is needed to determine the impact of high UCFB consumption on child micronutrient status and growth patterns throughout childhood.

## AUTHOR CONTRIBUTIONS

Alissa M. Pries and Elaine L. Ferguson conceptualized and designed the study with input from Guy‐Marino Hinnouho. Guy‐Marino Hinnouho analyzed the data with the help of Amy MacDougall and drafted the manuscript. All authors reviewed and provided input on the final article.

## CONFLICT OF INTEREST STATEMENT

The authors declare no conflict of interest.

## Data Availability

The data used for this study can be accessed by contacting Helen Keller Intl at data@hki.org.

## References

[mcn13485-bib-0001] Anderson, V. P. , Cornwall, J. , Jack, S. , & Gibson, R. S. (2008). Intakes from non‐breastmilk foods for stunted toddlers living in poor urban villages of Phnom Penh, Cambodia, are inadequate. Maternal & Child Nutrition, 4(2), 146–159. 10.1111/j.1740-8709.2007.00120.x 18336647 PMC6860648

[mcn13485-bib-0002] Anzman, S. L. , Rollins, B. Y. , & Birch, L. L. (2010). Parental influence on children's early eating environments and obesity risk: Implications for prevention. International Journal of Obesity, 34(7), 1116–1124. 10.1038/ijo.2010.43 20195285

[mcn13485-bib-0003] Beal, T. , White, J. M. , Arsenault, J. E. , Okronipa, H. , Hinnouho, G.‐M. , Murira, Z. , Torlesse, H. , & Garg, A. (2021). Micronutrient gaps during the complementary feeding period in south Asia: A comprehensive nutrient gap assessment. Nutrition Reviews, 79(Suppl 1), 26–34. 10.1093/nutrit/nuaa144 33693912 PMC7947968

[mcn13485-bib-0004] Birch, L. L. (1999). Development of food preferences. Annual Review of Nutrition, 19, 41–62. 10.1146/annurev.nutr.19.1.41 10448516

[mcn13485-bib-0005] Black, R. E. , Allen, L. H. , Bhutta, Z. A. , Caulfield, L. E. , De Onis, M. , Ezzati, M. , Mathers, C. , & Rivera, J. (2008). Maternal and child undernutrition: Global and regional exposures and health consequences. The Lancet, 371(9608), 243–260. 10.1016/S0140-6736(07)61690-0 18207566

[mcn13485-bib-0006] Coates, J. , Swindale, A. , & Bilinsky, P. (2007). Household food insecurity access scale (HFIAS) for measurement of household food access: Indicator guide (Version 3). FHI 360/FANTA.

[mcn13485-bib-0007] Cogill, B. (2003). Anthropometric indicators measurement guide: Food and nutrition technical assistance project. Academy for Educational Development.

[mcn13485-bib-0008] Cohen, J. (1960). A coefficient of agreement for nominal scales. Educational and Psychological Measurement, 20(1), 37–46. 10.1177/001316446002000104

[mcn13485-bib-0009] Costa, C. S. , Del‐Ponte, B. , Assunção, M. C. F. , & Santos, I. S. (2018). Consumption of ultra‐processed foods and body fat during childhood and adolescence: A systematic review. Public Health Nutrition, 21(1), 148–159. 10.1017/S1368980017001331 28676132 PMC10260745

[mcn13485-bib-0010] De Cosmi, V. , Scaglioni, S. , & Agostoni, C. (2017). Early taste experiences and later food choices. Nutrients, 9(2), 107. 10.3390/nu9020107 28165384 PMC5331538

[mcn13485-bib-0011] De Onis, M. , Garza, C. , Victora, C. G. , Onyango, A. W. , Frongillo, E. A. , & Martines, J. (2004). The WHO multicentre growth reference study: Planning, study design, and methodology. Food and Nutrition Bulletin, 25(1 Suppl), S15–S26. 10.1177/15648265040251S103 15069916

[mcn13485-bib-0012] Feeley, A. B. , Ndeye Coly, A. , Sy Gueye, N. Y. , Diop, E. I. , Pries, A. M. , Champeny, M. , Zehner, E. R. , & Huffman, S. L. (2016). Promotion and consumption of commercially produced foods among children: Situation analysis in an urban setting in Senegal. Maternal & Child Nutrition, 12(Suppl 2), 64–76. 10.1111/mcn.12304 PMC5071683 27061957

[mcn13485-bib-0013] Gibney, M. J. , Forde, C. G. , Mullally, D. , & Gibney, E. R. (2017). Ultra‐processed foods in human health: A critical appraisal. The American Journal of Clinical Nutrition, 106(3), 717–724. 10.3945/ajcn.117.160440 28793996

[mcn13485-bib-0014] Green, M. , Hadihardjono, D. N. , Pries, A. M. , Izwardy, D. , Zehner, E. , & Huffman, S. L. (2019). High proportions of children under 3 years of age consume commercially produced snack foods and sugar‐sweetened beverages in Bandung City, Indonesia. Maternal & Child Nutrition, 15(Suppl 4), e12764. 10.1111/mcn.12764 31225706 PMC6619027

[mcn13485-bib-0015] Healthy Ireland . (2018). Feeding your baby: Introducing family foods. Health Service Executive.

[mcn13485-bib-0016] Horta, B. L. , Loret de Mola, C. , & Victora, C. G. (2015). Breastfeeding and intelligence: A systematic review and meta‐analysis. Acta Paediatrica, 104(467), 14–19. 10.1111/apa.13139 26211556

[mcn13485-bib-0017] Huffman, S. L. , Piwoz, E. G. , Vosti, S. A. , & Dewey, K. G. (2014). Babies, soft drinks and snacks: A concern in low‐ and middle‐income countries? Maternal & Child Nutrition, 10(4), 562–574. 10.1111/mcn.12126 24847768 PMC4299489

[mcn13485-bib-0018] Jannat, K. , Luby, S. P. , Unicomb, L. , Rahman, M. , Winch, P. J. , Hossain, M. I. , & Stewart, C. P. (2020). Snack food consumption among Bangladeshi children, supplementary data from a large RCT. Maternal & Child Nutrition, 16(4), e12994. 10.1111/mcn.12994 32196968 PMC7507356

[mcn13485-bib-0019] Jimenez‐Cruz, A. , Bacardi‐Gascon, M. , Pichardo‐Osuna, A. , Mandujano‐Trujillo, Z. , & Castillo‐Ruiz, O. (2010). Infant and toddlers' feeding practices and obesity amongst low‐income families in Mexico. Asia Pacific Journal of Clinical Nutrition, 19(3), 316–323. 20805074

[mcn13485-bib-0020] Juul, F. , Parekh, N. , Martinez‐Steele, E. , Monteiro, C. A. , & Chang, V. W. (2022). Ultra‐processed food consumption among US adults from 2001 to 2018. The American Journal of Clinical Nutrition, 115(1), 211–221. 10.1093/ajcn/nqab305 34647997

[mcn13485-bib-0021] Karnopp, E. V. N. , Vaz, J. S. , Schafer, A. A. , Muniz, L. C. , Souza, R. L. V. , Santos, I. , Gigante, D. P. , & Assunção, M. C. F. (2017). Food consumption of children younger than 6 years according to the degree of food processing. Jornal de Pediatria, 93(1), 70–78. 10.1016/j.jped.2016.04.007 27393684

[mcn13485-bib-0071] Kelly, B. , Hebden, L. , King, L. , Xiao, Y. , Yu, Y. , He, G. , Li, L. , Zeng, L. , Hadi, H. , Karupaiah, T. , Hoe, N. S. , Noor, M. I. , Yoon, J. , & Kim, H. (2016). Children's exposure to food advertising on free-to-air television: An Asia-Pacific perspective. Health Promotion International, 31(1), 144–152. 10.1093/heapro/dau055 24997194

[mcn13485-bib-0022] Kimmons, J. E. , Dewey, K. G. , Haque, E. , Chakraborty, J. , Osendarp, S. J. M. , & Brown, K. H. (2005). Low nutrient intakes among infants in rural Bangladesh are attributable to low intake and micronutrient density of complementary foods. The Journal of Nutrition, 135(3), 444–451. 10.1093/jn/135.3.444 15735076

[mcn13485-bib-0023] Krumpal, I. (2013). Determinants of social desirability bias in sensitive surveys: A literature review. Quality & Quantity, 47(4), 2025–2047. 10.1007/s11135-011-9640-9

[mcn13485-bib-0024] Landis, J. R. , & Koch, G. G. (1977). The measurement of observer agreement for categorical data. Biometrics, 33(1), 159–174. 843571

[mcn13485-bib-0025] Lioret, S. , McNaughton, S. A. , Spence, A. C. , Crawford, D. , & Campbell, K. J. (2013). Tracking of dietary intakes in early childhood: The Melbourne InFANT program. European Journal of Clinical Nutrition, 67(3), 275–281. 10.1038/ejcn.2012.218 23321573 PMC5385208

[mcn13485-bib-0026] Luiten, C. M. , Steenhuis, I. H. , Eyles, H. , Ni Mhurchu, C. , & Waterlander, W. E. (2016). Ultra‐processed foods have the worst nutrient profile, yet they are the most available packaged products in a sample of New Zealand supermarkets—CORRIGENDUM. Public Health Nutrition, 19(3), 539. 10.1017/S1368980015002840 26419699 PMC10271151

[mcn13485-bib-0027] Luque, V. , Escribano, J. , Closa‐Monasterolo, R. , Zaragoza‐Jordana, M. , Ferré, N. , Grote, V. , Koletzko, B. , Totzauer, M. , Verduci, E. , ReDionigi, A. , Gruszfeld, D. , Socha, P. , Rousseaux, D. , Moretti, M. , Oddy, W. , & Ambrosini, G. L. (2018). Unhealthy dietary patterns established in infancy track to mid‐childhood: The EU childhood obesity project. The Journal of Nutrition, 148(5), 752–759. 10.1093/jn/nxy025 29982656

[mcn13485-bib-0028] Malik, V. S. , Pan, A. , Willett, W. C. , & Hu, F. B. (2013). Sugar‐sweetened beverages and weight gain in children and adults: A systematic review and meta‐analysis. The American Journal of Clinical Nutrition, 98(4), 1084–1102. 10.3945/ajcn.113.058362 23966427 PMC3778861

[mcn13485-bib-0029] Martins, A. P. B. , Levy, R. B. , Claro, R. M. , Moubarac, J.‐C. , & Monteiro, C. A. (2013). Participacao crescente de produtos ultraprocessados na dieta brasileira (1987‐2009). Revista de saúde pública, 47(4), 656–665. 10.1590/S0034-8910.2013047004968 24346675

[mcn13485-bib-0030] Ministerio de Salud (MINSA) Panama . (2018). *Guías alimentarias para los menores de 2 años de Panamá*.

[mcn13485-bib-0031] Monteiro, C. A. , Levy, R. B. , Claro, R. M. , de Castro, I. R. R. , & Cannon, G. (2010). Increasing consumption of ultra‐processed foods and likely impact on human health: Evidence from Brazil. Public Health Nutrition, 14(1), 5–13. 10.1017/S1368980010003241 21211100

[mcn13485-bib-0032] Monteiro, C. A. , Moubarac, J.‐C. , Cannon, G. , Ng, S. W. , & Popkin, B. (2013). Ultra‐processed products are becoming dominant in the global food system. Obesity Reviews, 14(Suppl 2), 21–28. 10.1111/obr.12107 24102801

[mcn13485-bib-0033] Nakphong, M. K. , & Beltrán‐Sánchez, H. (2021). Socio‐economic status and the double burden of malnutrition in Cambodia between 2000 and 2014: Overweight mothers and stunted children. Public Health Nutrition, 24(7), 1806–1817. 10.1017/S1368980021000689 33632364 PMC8094435

[mcn13485-bib-0034] National Institute of Statistics (NIS) [Cambodia], Ministry of Health (MoH) [Cambodia], & ICF . (2022). *Cambodia demographic and health survey 2021–22 key indicators report*. Phnom Penh, Cambodia, and Rockville: NIS, MoH, and ICF.

[mcn13485-bib-0035] Neri, D. , Steele, E. M. , Khandpur, N. , Cediel, G. , Zapata, M. E. , Rauber, F. , Marrón‐Ponce, J. A. , Machado, P. , Costa Louzada, M. L. , Andrade, G. C. , Batis, C. , Babio, N. , Salas‐Salvadó, J. , Millett, C. , Monteiro, C. A. , & Levy, R. B. , NOVA Multi‐Country Study Group on Ultra‐Processed Foods, Diet Quality and Human Health . (2022). Ultraprocessed food consumption and dietary nutrient profiles associated with obesity: A multicountry study of children and adolescents. Obesity Reviews, 23(S1), e13387. 10.1111/obr.13387 34889015

[mcn13485-bib-0036] Nogueira, M. B. , Mazzucchetti, L. , Mosquera, P. S. , Cardoso, M. A. , & Malta, M. B. (2022). Consumption of ultra‐processed foods during the first year of life and associated factors in Cruzeiro do Sul, Brazil. Ciencia & Saude Coletiva, 27(2), 725–736. 10.1590/1413-81232022272.47072020 35137827

[mcn13485-bib-0037] Pagliai, G. , Dinu, M. , Madarena, M. P. , Bonaccio, M. , Iacoviello, L. , & Sofi, F. (2021). Consumption of ultra‐processed foods and health status: A systematic review and meta‐analysis. British Journal of Nutrition, 125(3), 308–318. 10.1017/S0007114520002688 32792031 PMC7844609

[mcn13485-bib-0038] Park, S. , Lin, M. , Onufrak, S. , & Li, R. (2015). Association of sugar‐sweetened beverage intake during infancy with dental caries in 6‐year‐olds. Clinical Nutrition Research, 4(1), 9–17. 10.7762/cnr.2015.4.1.9 25713788 PMC4337927

[mcn13485-bib-0039] Popkin, B. M. , Adair, L. S. , & Ng, S. W. (2012). Global nutrition transition and the pandemic of obesity in developing countries. Nutrition Reviews, 70(1), 3–21. 10.1111/j.1753-4887.2011.00456.x 22221213 PMC3257829

[mcn13485-bib-0040] Pries, A. M. , Filteau, S. , & Ferguson, E. L. (2019). Snack food and beverage consumption and young child nutrition in low‐and middle‐income countries: A systematic review. Maternal & Child Nutrition, 15, e12729. 31225715 10.1111/mcn.12729 PMC6618154

[mcn13485-bib-0041] Pries, A. M. , Huffman, S. L. , Champeny, M. , Adhikary, I. , Benjamin, M. , Coly, A. N. , Diop, E. H. I. , Mengkheang, K. , Sy, N. Y. , & Dhungel, S. (2017). Consumption of commercially produced snack foods and sugar‐sweetened beverages during the complementary feeding period in four African and Asian urban contexts. Maternal & Child Nutrition, 13, e12412. 29032629 10.1111/mcn.12412 PMC6865897

[mcn13485-bib-0042] Pries, A. M. , Huffman, S. L. , Mengkheang, K. , Kroeun, H. , Champeny, M. , Roberts, M. , & Zehner, E. (2016). High use of commercial food products among infants and young children and promotions for these products in Cambodia. Maternal & Child Nutrition, 12(Suppl 2), 52–63. 10.1111/mcn.12270 27061956 PMC5021124

[mcn13485-bib-0043] Pries, A. M. , Rehman, A. M. , Filteau, S. , Sharma, N. , Upadhyay, A. , & Ferguson, E. L. (2019). Unhealthy snack food and beverage consumption is associated with lower dietary adequacy and length‐for‐age z‐scores among 12‐23‐month‐olds in Kathmandu Valley, Nepal. The Journal of Nutrition, 149(10), 1843–1851. 10.1093/jn/nxz140 31309223 PMC6768809

[mcn13485-bib-0044] Rahman, M. J. , Nizame, F. A. , Nuruzzaman, M. , Akand, F. , Islam, M. A. , Parvez, S. M. , Stewart, C. P. , Unicomb, L. , Luby, S. P. , & Winch, P. J. (2016). Toward a scalable and sustainable intervention for complementary food safety. Food and Nutrition Bulletin, 37(2), 186–201. 10.1177/0379572116631641 26944506

[mcn13485-bib-0045] Reynolds, W. M. (1982). Development of reliable and valid short forms of the marlowe‐crowne social desirability scale. Journal of Clinical Psychology, 38, 119–125. 10.1002/1097-4679(198201)38:13.0.CO;2-I

[mcn13485-bib-0046] Rose, C. M. , Birch, L. L. , & Savage, J. S. (2017). Dietary patterns in infancy are associated with child diet and weight outcomes at 6 years. International Journal of Obesity, 41(5), 783–788. 10.1038/ijo.2017.27 28133360

[mcn13485-bib-0047] Sadeghirad, B. , Duhaney, T. , Motaghipisheh, S. , Campbell, N. R. C. , & Johnston, B. C. (2016). Influence of unhealthy food and beverage marketing on children's dietary intake and preference: a systematic review and meta‐analysis of randomized trials: Meta‐analysis of unhealthy food and beverage marketing. Obesity Reviews, 17(10), 945–959. 10.1111/obr.12445 27427474

[mcn13485-bib-0048] Schwartz, C. , Scholtens, P. A. , Lalanne, A. , Weenen, H. , & Nicklaus, S. (2011). Development of healthy eating habits early in life. Review of recent evidence and selected guidelines. Appetite, 57(3), 796–807. 10.1016/j.appet.2011.05.316 21651929

[mcn13485-bib-0049] Sharma, N. , Ferguson, E. L. , Upadhyay, A. , Zehner, E. , Filteau, S. , & Pries, A. M. (2019). Perceptions of commercial snack food and beverages for infant and young child feeding: A mixed‐methods study among caregivers in Kathmandu Valley, Nepal. Maternal & Child Nutrition, 15, e12711. 31225712 10.1111/mcn.12711 PMC7198114

[mcn13485-bib-0050] Som, S. , Prak, S. , Laillou, A. , Gauthier, L. , Berger, J. , Poirot, E. , & Wieringa, F. (2018). Diets and feeding practices during the first 1000 days window in the Phnom Penh and north eastern districts of Cambodia. Nutrients, 10(4), 500. 10.3390/nu10040500 29670006 PMC5946285

[mcn13485-bib-0051] Stein, L. J. , Cowart, B. J. , & Beauchamp, G. K. (2012). The development of salty taste acceptance is related to dietary experience in human infants: A prospective study. The American Journal of Clinical Nutrition, 95(1), 123–129. 10.3945/ajcn.111.014282 22189260 PMC3238456

[mcn13485-bib-0052] Turton, B. , Chher, T. , Hak, S. , Sokal‐Gutierrez, K. , Lopez Peralta, D. , Laillou, A. , & Singh, A. (2022). Associations between dental caries and ponderal growth in children: A Cambodian study. Journal of Global Health, 12, 04046. 10.7189/jogh.12.04046 35713031 PMC9204672

[mcn13485-bib-0053] Twisk, J. W. R. , Kemper, H. C. G. , van Mechelen, W. , & Post, G. B. (1997). Tracking of risk factors for coronary heart disease over a 14‐year period: A comparison between lifestyle and biologic risk factors with data from the Amsterdam Growth and Health Study. American Journal of Epidemiology, 145(10), 888–898. 10.1093/oxfordjournals.aje.a009048 9149660

[mcn13485-bib-0054] U.S. Department of Agriculture & U.S. Department of Health and Human Services . (2020). Dietary guidelines for Americans, 2020–2025. 9th ed. DietaryGuidelines.gov.

[mcn13485-bib-0055] Ventura, A. K. , & Mennella, J. A. (2011). Innate and learned preferences for sweet taste during childhood. Current Opinion in Clinical Nutrition and Metabolic Care, 14(4), 379–384. 10.1097/MCO.0b013e328346df65 21508837 PMC12974590

[mcn13485-bib-0056] Victora, C. G. , Bahl, R. , Barros, A. J. D. , França, G. V. A. , Horton, S. , Krasevec, J. , Murch, S. , Sankar, M. J. , Walker, N. , & Rollins, N. C. , Lancet Breastfeeding Series Group . (2016). Breastfeeding in the 21st century: Epidemiology, mechanisms, and lifelong effect. The Lancet, 387(10017), 475–490. 10.1016/S0140-6736(15)01024-7 26869575

[mcn13485-bib-0057] Vyas, S. , & Kumaranayake, L. (2006). Constructing socio‐economic status indices: How to use principal components analysis. Health Policy and Planning, 21(6), 459–468. 10.1093/heapol/czl029 17030551

[mcn13485-bib-0058] Wang, L. , Martínez Steele, E. , Du, M. , Pomeranz, J. L. , O'Connor, L. E. , Herrick, K. A. , Luo, H. , Zhang, X. , Mozaffarian, D. , & Zhang, F. F. (2021). Trends in consumption of ultraprocessed foods among US youths aged 2‐19 years, 1999‐2018. Journal of the American Medical Association, 326(6), 519–530. 10.1001/jama.2021.10238 34374722 PMC8356071

[mcn13485-bib-0059] WHO . (2010). *Set of recommendations on the marketing of foods and non‐alcoholic beverages to children for assessing infant and young child feeding practices*. WHO Press.

[mcn13485-bib-0060] WHO Multicentre Growth Reference Study Group . (2006). WHO child growth standards based on length/height, weight and age. Acta Paediatrica, 450(Suppl), 76–85. 10.1111/j.1651-2227.2006.tb02378.x 16817681

[mcn13485-bib-0061] World Health Organization . (1998). Complementary feeding of young children in developing countries: A review of current scientific knowledge. *WHO/NUT/98.1*. WHO IRIS. https://apps.who.int/iris/handle/10665/65932

[mcn13485-bib-0062] World Health Organization . 2020, August 7. *Public health surveillance for COVID‐19: Interim guidance*. World Health Organization.

[mcn13485-bib-0063] World Health Organization & UNICEF (Eds.). (2003). Global strategy for infant and young child feeding. WHO.

[mcn13485-bib-0064] World Health Organization & UNICEF (Eds.). (2021). Indicators for assessing infant and young child feeding practices: Definitions and measurement methods. World Health Organization. https://apps.who.int/iris/handle/10665/340706

